# Improving the Safety of Tolerance Induction: Chimerism and Cellular Co-Treatment Strategies Applied to Vascularized Composite Allografts

**DOI:** 10.1155/2012/107901

**Published:** 2012-10-22

**Authors:** Wei-Chao Huang, Jeng-Yee Lin, Christopher Glenn Wallace, Fu-Chan Wei, Shuen-Kuei Liao

**Affiliations:** ^1^Division of Plastic and Reconstructive Surgery, Tzu Chi General Hospital, New Taipei 231, Taiwan; ^2^Graduate Institute of Clinical Medical Sciences, College of Medicine, Chang Gung University, Taoyuan 333, Taiwan; ^3^Division of Plastic Surgery, Department of Surgery, Taipei Medical University Hospital, Taipei 110, Taiwan; ^4^Department of Plastic and Reconstructive Surgery, Chang Gung Memorial Hospital, Taoyuan 333, Taiwan; ^5^Cancer Immunotherapy Program, Cancer Center, Taipei Medical University Hospital and Center of Excellence for Cancer Research, Taipei Medical University, 252 Wu-Hsing Street, Taipei 110, Taiwan

## Abstract

Although vascularized composite allografts (VCAs) have been performed clinically for a variety of indications, potential complications from long-term immunosuppression and graft-versus-host disease remain important barriers to widespread applications. Recently it has been demonstrated that VCAs incorporating a vascularized long bone in a rat model provide concurrent vascularized bone marrow transplantation that, itself, functions to establish hematopoietic chimerism and donor-specific tolerance following non-myeloablative conditioning of recipients. Advances such as this, which aim to improve the safety profile of tolerance induction, will help usher in an era of wider clinical VCA application for nonlife-saving reconstructions.

## 1. Introduction 

It has become increasingly appreciated that vascularized composite allografts (VCAs) can, in select patients, provide the best reconstructive alternative for complex losses, such as of total larynx [1], total hand/forearm [2–4], and composite subtotal facial deficits [5], which have until now been essentially non-reconstructible. The problems that hold VCA back from completely revolutionizing reconstructive surgery are predominantly immunological [6]. VCAs contain highly immunogenic (e.g., skin) and immunocompetent (e.g., marrow and blood) tissues that, when revascularized by the recipient vessels, instigate a battle of immune systems leading to transplant rejection and/or graft-versus-host disease (GVHD) unless intricately controlled exogenously by immunosuppression. If robust tolerance between VCA and host could be achieved repeatedly and predictably without causing significant host morbidity, the gates could be opened for a reconstructive paradigm shift led by VCA. The literature addressing the concepts highlighted in this paper is immense and it is not intended that it be exhaustively reviewed. Instead, we provide an overview of the current trends in chimerism-based tolerance research and focus particularly upon recent cellular co-treatment strategies that have real potential for translation to the clinical setting. 

## 2. Current Immunosuppression to Avoid VCA Rejection

VCA became a clinical reality using immunosuppression regimens imported from solid organ transplantation [6]. Transplant recipients require life-long immunosuppression, commencing with induction followed by maintenance therapy. Maintenance may be interspersed with rescue therapies if episodes of acute rejection occur. The goals of tailoring immunosuppression according to recipient and transplant response are to prevent acute and chronic rejection, to minimize the toxicities of immunosuppressants and the rates of infection and malignancy, and to maximize patient and graft survival without GVHD [7]. 

Induction therapy for clinical VCA consists of antilymphocyte antibody or anti-T-cell therapy (using blocking or deleting agents) administered parenterally for a short course immediately posttransplant [8]. The underlying rationale for using these agents is their potent anti-T-cell immunosuppressive properties. Induction therapy is used in conjunction with maintenance agents to minimize early rejection episodes [8]. Induction agents include OKT3, anti-thymocyte globulin (ATG), daclizumab, and basiliximab [9]. Whilst induction therapies may be used in an attempt to induce a tolerogenic effect to donor alloantigen, the experimental evidence indicates that tolerance is unlikely to follow if such treatments are used alone [10, 11]. Maintenance agents used in VCA include corticosteroids (prednisone), cyclosporine, tacrolimus, azathioprine, mycophenolate mofetil (MMF), and sirolimus. 

In the early 1990s cyclosporine-AZA steroid-based regimens were used in a series of clinical CTAs to reconstruct nerves, tendons, muscle, bone, joint, and laryngeal defects [6]. In 1997, tacrolimus/MMF/prednisone-based regimens developed by the Louisville group were used successfully to prevent VCA rejection, especially of the skin, while causing minimal systemic toxicity in a preclinical swine forelimb model [6]. This tacrolimus/MMF/prednisone-based combination therapy, similar to that used for solid organ transplants, was utilized thereafter for hand transplantations.

Despite rigorous immunosuppression, however, transplants are not necessarily completely spared from acute rejection episodes and, additionally, chronic rejection is likely deleterious to the long-term function of VCAs [12]. Hence, subjecting patients to life-long immunosuppression regimens that do not completely control against acute rejection episodes nor against chronic functional decline of a non-life-saving reconstructive transplantation remains ethically problematic.

## 3. Graft-Versus-Host Disease in VCA Recipients

A critical feature of VCAs that distinguish them entirely from solid organ transplants is the massive lymphoid armament of donor immunocompetent cells from marrow and lymph nodes that attempt to reject the recipient and may cause GVHD. T cells have been identified as the most important effector cellular subset in this reaction although other cell populations may also participate [13, 14]. 

In attempts to relieve the risks of GVHD, attention has recently been paid to the preparation of more tolerogenic cell populations, notably plasmacytoid dendritic cells (DCs) [15], hematopoietic stem cells (HSCs), and mesenchymal stem cells (MSCs) [16]. Infusion of MSCs from a third-party reduced GVHD in allogeneic bone marrow transplantation (BMT) in leukemia patients [16]. MSCs have also been shown to facilitate the induction of mixed hematopoietic chimerism and islet allograft tolerance without GVHD in rats [17].

Additionally, removal of mature T cells from the transplanted bone marrow graft has prevented GVHD effectively in mice, rats, and humans [18]. Depletion of both *αβ* T cells and *γδ* T cells from the donor marrow inoculums prevented GVHD, implicating a role for either or both types of T cells as effectors in GVHD [19]. Importantly, this approach to T-cell depletion does not remove facilitating cells (FCs), nor does it compromise engraftment [18]. The phenotype of FCs is similar to that of plasmacytoid DCs, which are known to mediate antigen-specific tolerance and induce CD4^+^ as well as CD8^+^ regulating T cells *in vitro *[15, 20, 21].

To prevent the occurrence of GVHD in VCAs, T-cell depletion of grafts has been explored in an attempt to lower transplant-related mortality [22]. Selective techniques to prevent GVHD without causing immune deficiency and increased infection provoked by systemic T-cell depletion can be achieved by preirradiating the transferred hind-limb with a lethal dose [23] or by lymphadenectomy [24]. However, irradiation of the donor tissue may increase graft failure, and lymphadenectomy is a time-consuming procedure that has its own complications. These two methods have limited potential for clinical practice. An alternative promising approach is graft perfusion with anti-T cell receptor (TCR) monoclonal antibody (mAb) [22]. This approach immunomodulates the vascularized bone graft to reduce GVHD after VCA and concomitantly promotes long-term donor-specific tolerance in the host.

## 4. Tolerogenicity of Vascularized Bone Grafts within VCA 

Ideally, VCA recipients would be treated with an effective antirejection therapy that could be tapered quickly to a maintenance dose and then stopped to reduce immunosuppression-related complications [25]. Complete withdrawal of immunosuppression would be possible if donor-specific tolerance develops and the functional recovery of the transplant would not be jeopardized by its withdrawal, hence providing solutions to both chronic rejection and immunosuppressive drug toxicity. 

The induction and maintenance of tolerance to allotransplants constitutes an active process involving multiple mechanisms that work cooperatively to prevent graft rejection [26]. The creation of hematopoietic chimerism through BMT remains the most stable method for inducing transplantation tolerance [27]. Chimerism refers to a state in which two genetically different hematopoietic systems are harmoniously present and functioning in one organism [28]. Successful achievement of mixed chimerism tolerizes T cells and B cells to both donor and host tissues [29]. Whilst chimerism is associated with the induction of tolerance, our research has indicated that it depletes after several months and yet allograft tolerance is maintained [30, 31]. Therefore, cell migration and chimerism are believed an invariable early event in graft acceptance [32]. Irradiation of the recipient promotes cell migration and engraftment of the infused donor HSCs. Conventional BMT can induce chimerism leading to tolerance, but involves the following sequence: host conditioning with irradiation, donor BMT, characterization of chimerism by flow cytometry (at 28 days), and allotransplantation [27]. This 28-day period has been considered a requirement for engraftment and repopulation of the donor bone marrow cells in the host [27]. Allotransplantation performed before successful engraftment of donor bone marrow may interfere with the establishment of tolerance [27]. To overcome this 28-day delay, operational tolerance through cyclophosphamide, anti-CD2 mAb, or thymic irradiation was successfully induced in four out of five patients with end-stage renal disease receiving bone marrow transplantation in addition to a kidney from related living donors [31]. Despite withdrawing immunosuppression, renal function remained normal for up to 5.3 years after transplantation. In addition, immediate tolerance to skin graft was observed in rodent models using BMT and costimulatory blockade with reduced myeloablative host conditioning. However, exposing an otherwise healthy patient with a non-life-threatening functional deficit to irradiation and the creation of chimerism is ethically difficult. 

A unique feature of some VCAs (e.g., hand/forearm, knee) that has been exploited recently is the presence of a vascularized bone marrow transplant (VBMT) component within the incorporated long bone(s). This instantly and continuously produces bone marrow cells once transplanted and directly provides the niche for reconstitution of HSCs [30]. Bone marrow additionally contains stromal cells, including fibroblasts, adipocytes, endothelial cells, and osteoblasts, derived from MSCs that are known to influence the HSC microenvironment [33]. Stromal cells appear to be capable of supporting HSCs and progenitor cells *in vitro *and* in vivo* and a stromal microenvironment is essential for the proliferation and differentiation of hematopoietic progenitors [33]. 

Early engraftment and reconstitution of multiple hematopoietic lineages may allow for instant establishment of chimerism and earlier tolerance to VCAs. VBMT within VCA, and not conventional BMT, was critical to the long-term establishment of chimerism and tolerance under partially myeloablative conditioning and tacrolimus-based treatment [30]. It can be concluded therefore that the VBMT within VCA provides critical signaling and modulatory functions that initiate tolerance induction. Importantly, this study showed that it was therefore possible to overcome the 28-day delay to engraftment because the VBMT within VCA could, under the correct conditions, induce mixed chimerism and tolerance simultaneously. 

## 5. Other Methods of Tolerance Induction for VCA

### 5.1. T-Cell Depletion

T cell depletion prior to VCA transplantation followed by T-cell repopulation after allotransplantation has been associated with allograft acceptance in animal models and in humans [34, 35]. Nonspecific T-cell depletion (lymphodepletion) medications such as ATG [36, 37], Orthoclone mAb OKT3 [38], and humanized Campath-1H CD52 mAb [39] are frequently used clinically for induction therapy before transplantation for prevention and treatment of acute rejection episodes. Selective T-cell inhibition using *αβ*-TCR monoclonal antibodies combined with cyclosporine has been associated with robust mixed chimerism and long-term VCA survival in animal models [40, 41].

### 5.2. Costimulation Blockade

Full T-cell function requires binding of the TCR to MHC molecules on DCs or other antigen-presenting cells (APCs), alongside profoundly influential co-stimulatory signals, examples of which include the CD154 and CD28 interactions with CD40 and B7 ligands, respectively [42, 43]. Without adequate co-stimulation, T cells may undergo apoptosis, inactivation, or anergy [42]. Selective interference with these co-stimulators is therefore an attractive way to influence the behavior of T cells that encounter specific antigens.

Costimulation blockade, with or without BMT, using anti-CD154 (anti-CD40L) mAb alone or together with CTLA-4 Ig (CTLA-4 immunoglobulin fusion protein) has been reported to prolong survival or induce tolerance to VCA in rodents and large animals [42–48]. Various mechanisms have been proposed in prolongation of solid organ allograft survival using co-stimulatory blockade, including anergy, suppression, and deletion. The most recent data suggest that deletion of peripheral alloreactive T cells has a major role in the establishment of mixed chimerism using co-stimulatory blockade. The use of co-stimulatory blocking reagents in BMT protocols can facilitate the induction of mixed chimerism while markedly reducing the potential toxicity of conditioning using total body irradiation (TBI), thymic irradiation or host T-cell depletion. CTLA-4 Ig is a biological agent consisting of the extracellular domain of CD152 fused to the Fc region of IgG1. As CTLA-4 Ig is potentially tolerogenic through costimulatory blockade it has been explored extensively in transplantation. It was subsequently deduced in mice that administration of CTLA-4 Ig resulted in the induction of indoleamine 2,3-deoxygenase (IDO) in professional APCs, like DCs [49]. IDO is induced during inflammation by IFN-*γ* [50] and other proinflammatory cytokines and acts to deplete the local microenvironment of the essential amino acid, tryptophan. The resulting low levels of extracellular tryptophan act as a signal to inhibit T-cell proliferation.

### 5.3. *In Vitro* Manipulation of Donor Dendritic Cells

Dendritic cells (DCs) are considered the most potent class of APCs, the mature form of which express MHC class I and II molecules. They bear a variety of costimulatory signal molecules such as CD40, CD80, and CD86 that are responsible for presenting antigen to T cells for T-cell activation. The majority of DCs are functionally immature and therefore incapable of presenting donor antigen to T-cells efficiently, leading to disabled T-cell activation [51]. This has been exploited to support VCA tolerance induction by, prior to transplantation, infusing pharmacologically stabilized immature DCs that have been pulsed with donor antigen [52, 53]. DCs are also noted to induce tolerogenic regulatory T cells (T_reg_) that in turn promote allograft tolerance [52, 53]. The administration of recipient-derived DCs prolonged VCA survival in animal models but DCs *per se *were unable to induce transplantation tolerance to VCA [54, 55]. 

### 5.4. Mesenchymal Stem Cells

Another intriguing recent finding that requires further research has been the ability of infused syngeneic, allogeneic, and even third-party adipose-derived MSCs to promote long-term survival of VCA between fully MHC-mismatched rats without causing GVHD [56]. Clinically, MSCs could modulate immune responses and ameliorate GVHD after hematopoietic-stem-cell transplantation [57]. How exactly MSCs modulate the immune response is not completely understood. However, some mechanisms involved in such modulation have been proposed, which include the expression of HLA-G molecules, direct interactions with DCs preventing them from differentiation and maturation, and modulation of the expression of cytokines/factors such as IL-10, TGF-*β*, IDO, TNF-*α* and INF-*γ* [58].  Co-treatment of MSCs with BM cells before VCA transplantation with low-dose (3 Gy) irradiation conditioning significantly prolonged allograft survival without GVHD as compared to animals that did not receive MSC co-treatment [59].

### 5.5. Regulatory T-Cell Therapy

Further study into our model [60] demonstrated that the T_reg_/CD4^+^ ratio in the peripheral blood of VCA-accepting chimera was negatively correlated with mixed chimerism levels. This suggests a significant role for T_reg_ in maintaining VCA tolerance when mixed chimerism is less robust. A body of literature reports that T_regs_ have exceptional therapeutic effects on autoimmune diseases [61], organ transplantation [62, 63], and GVHD models [64, 65], but do not induce skin graft tolerance across full MHC barriers when utilized alone [66, 67]. Interestingly, combined therapy with T_regs_ and allogeneic BMT has been reported to achieve durable mixed chimerism and long-term tolerance to nonvascularized skin allografts without cytoreductive conditioning in mice [68]. This approach was recently tested in rats in our laboratories [69]. A combination of T_regs_ prepared from the recipient strain and VBMT treatment, with a short course conditioning of recipients with costimulation blockade and rapamycin, led to long-term multilineage hematopoietic mixed chimerism (12–18%) and long-term donor-specific tolerance to VCA (89% acceptance rate) without GVHD. Neither stable mixed chimerism nor VCA acceptance was observed in recipients without T_reg_ treatment. Interestingly, FoxP3^+^  T_reg_ cells infiltrated VCA near the donor/allograft tissue junction in VCA-accepting chimera, further suggesting an importance for them in permitting long-term VCA survival [68–70]. Of note is that the FoxP3^+^T_reg_ cells found in the donor/allograft tissue junction were mostly of recipient origin (JY Lin and SK Liao, unpublished). Nevertheless, the question as to whether they belong to natural or induced T_regs_ remains to be determined. The presence of T_regs_ in the donor skin of hand allotransplantation recipients has been demonstrated as has increased FoxP3 expression during rejection episodes at a later time point after transplantation [71, 72]. These findings also support that T_reg_ may play a significant role in maintaining allograft acceptance and prevent allograft rejection by downregulation of donor-reactive effectors infiltrating the donor graft. However, the clinical relevance and exact mechanism of immunomodulation by T_regs_ in tolerance induction to VCA remains unclear and further investigations are warranted. 

Allogeneic T_reg_, third-party T_reg_ [73, 74] and MSC preparations [75, 76] are just some examples of how immunosuppressive functions from diverse cellular sources have been exploited to ease tolerance induction in recent years. Encouragingly, these biologicals can be cryopreserved in liquid nitrogen and hence could be made available in large quantities when required to facilitate VCA acceptance in the future. So far, stable mixed chimerism, which has been successfully established in rodent models for tolerance induction to VCA, has not been observed in the majority of clinical VCA transplantations without recipient conditioning, thus making translation of such protocols into clinical application less likely. The major hindrance to widespread use of mixed allogeneic chimerism as a strategy to induce VCA tolerance in the clinical setting will be the requirement for recipient conditioning and the risk of GVHD. We believe that combination approaches, such as our noncytoreductive T_reg_-VBMT protocol or tailored cell-based therapies alongside low-dose immunosuppression, may have improved potential for clinical application in VCA transplantation. Current widely researched methods of tolerance induction are summarized in a chronological order in Table 1.

## 6. Conclusions

Encouraging results continue to be obtained from VCA investigations in rats focusing on developing nonmyeloablative methods of establishing mixed chimerism. So far there has been a lack of evidence showing the presence of mixed chimerism or donor-specific unresponsiveness in all clinical VCA transplantation recipients (including 53 hand transplantations that inherently included VBMT and one face transplantation with bone marrow infusion), suggesting stable mixed chimerism cannot be induced without host conditioning in humans. Clinical VCA transplantations will benefit from further investigations searching for tolerance protocols involving less toxic host conditioning. Protocols that have promise incorporate co-treatment strategies, including the use of cell-based therapies involving VBMT, T_reg_, and/or MSCs. Whilst progress is made in improving the safety of tolerance induction, it will become increasingly important to develop recipient monitoring measures that can accurately reflect the success or failure of tolerance induction in these animal models. Such tests should ultimately be considered an integral part of tolerizing protocols to be used clinically for VCA. 

## Figures and Tables

**Table 1:** Methods of tolerance induction for VCA listed chronologically.

Methods	Induction pathway
Chimerism	Bone marrow transplantation
	Vascularized bone grafts
T cell depletion	ATG, OKT3 mAb, Campath-1H (CD52) mAb, *αβ* TCR/*γδ* TCR mAb
Costimulation blockade	CD154, CD28, and/or CD40 mAbs, B7 ligand, CTLA-4 Ig (fusion protein)
Donor dendritic cell	*In vitro *manipulation followed by intravenous infusion
Mesenchymal stem cell	*Ex vivo* expansion followed by intravenous infusion
Regulatory T cell	*In vivo* induction followed by intravenous infusion
